# Transforming academia for equity: implementation at the University of Memphis

**DOI:** 10.3389/fpubh.2026.1690688

**Published:** 2026-05-22

**Authors:** Marian Levy, Debra Bartelli, Fawaz Mzayek, Xinhua Yu, Stephen Zanskas, Demetria Frank, Clarion Harris, Andrea Jacobo, Kimberly Harris, Sandra Silva

**Affiliations:** 1 School of Public Health, University of Memphis, Memphis, TN, United States; 2 College of Education, University of Memphis, Memphis, TN, United States; 3 DD Consulting LLC, Houston, TX, United States; 4 Upskill Mid-South, Center for Community Research and Evaluation, University of Memphis, Memphis, TN, United States; 5 Division of Research and Innovation, University of Memphis, Memphis, TN, United States; 6 Change Matrix LLC, Denver, CO, United States

**Keywords:** academia, academic equity, academic public health, belonging, inclusion

## Abstract

In 2022, with funding from the Robert Wood Johnson Foundation’s (RWJF) *Transforming Academia for Equity* grant program, and in partnership with the Association of Schools and Programs in Public Health (ASPPH) the University of Memphis (UofM) School of Public Health (SPH) began a systematic, coordinated process to develop and implement a strategic plan of action that provides an inclusive culture and supports historically excluded and minoritized scholars and students. We formed a guiding team that consisted of key SPH leaders and experienced campus administrators from other UofM colleges (Law, Education, and Communication and Fine Arts). Using a data-driven approach (climate surveys, listening sessions, and focus groups), we identified priorities and developed activities and training sessions that responded to what we heard. These activities and sessions included faculty/staff workshops and a graduate student mentoring program. Importantly, we also identified salient processes and metrics that have been embedded in the School of Public Health’s new Strategic Plan that will support sustainability.

## Introduction

Creating equitable, inclusive, and supportive academic environments is essential to advancing excellence in public health education and research. Yet many schools of public health continue to face persistent structural barriers that limit the success, retention, and advancement of scholars from historically excluded and minoritized groups (1–4). In 2022, the University of Memphis School of Public Health (UofM SPH) was selected as one of seven institutions nationwide to receive funding from the Robert Wood Johnson Foundation’s *Transforming Academia for Equity* initiative. According to the Call for Proposals, the initiative was intended to reshape systems and structures within academia to better support “historically excluded scholars” both professionally and personally and to create inclusive space “where equity, diversity, and inclusivity (EDI) are valued and effectively integrated in the institutional policies, culture, and behaviors” (5). This support provided an opportunity to undertake a coordinated, school-wide effort to examine existing structures, identify barriers to equity, and implement sustainable strategies that strengthen belonging and academic success for students, faculty, and staff.

The UofM SPH entered this initiative with three interconnected goals:

1. Strengthen recruitment and retention of historically excluded faculty;
2. Examine and address inequities in Tenure and Promotion (T&P) policies and processes; and.
3. Develop pathways and mentorship structures for first-generation and historically excluded graduate students, particularly those who aspire to doctoral training and academic careers.

These goals were informed by the School’s demographic context. While the SPH student body included a substantial proportion of minoritized and first-generation students, faculty diversity remained limited, creating a mismatch between the identities of students and those who teach and mentor them. Understanding how this environment shapes experiences of inclusion, belonging, and academic opportunity was a central motivation for the project.

To guide this work, the SPH established a multidisciplinary Guiding Team composed of SPH faculty, graduate students, staff, and “inclusion champions” from the Colleges of Law, Education, and Communication and Fine Arts—colleagues recognized for their expertise in building equitable academic infrastructures. With technical assistance from Change Matrix’s Adaptive Change Specialists, the team implemented a multi-method, data-driven process that included a school-wide climate survey, listening sessions with students, faculty, staff, and alumni, and focus groups examining T&P barriers for historically excluded faculty across campus.

Findings from these assessments informed a series of targeted actions, including faculty and staff inclusion workshops, development of a School-wide Community Agreement, and creation of the Academic Equity Fellowship (AEF) Program to support first-generation and historically excluded graduate students. Over the three-year funding period, these efforts contributed to measurable improvements in perceptions of inclusion and helped embed principles of belonging into the SPH’s strategic plan, orientation processes, and ongoing professional development structures.

This manuscript describes the implementation, outcomes, and lessons learned from the SPH’s participation in *Transforming Academia for Equity*. By detailing our processes, challenges, and strategies for sustainability, we aim to provide a model that other institutions may adapt as they work to advance equity within academic public health. In this manuscript, we use the term “historically excluded” to refer broadly to groups that have been systematically marginalized in academic public health, including those often described as underrepresented or minoritized.

## Materials and methods

### Guiding team formation and structure

To lead the *Transforming Academia for Equity Initiative*, the SPH established a 10-member Guiding Team representing multiple roles and perspectives within the SPH and University. Members included UofM SPH administrators (Associate Dean, Doctoral Coordinator, Faculty Senator); cross-disciplinary faculty (representing epidemiology, behavioral sciences, environmental health); graduate student representatives. To expand the initiative’s capacity and draw upon existing institutional expertise, the team also included three “inclusion champions” from the Colleges of Law, Education, and Communication & Fine Arts. These colleagues were selected based on their demonstrated leadership in developing equitable academic infrastructures, prior experience implementing inclusive pedagogical and administrative practices, and their roles in advancing diversity-related initiatives within their respective colleges. Their participation ensured that the SPH would benefit from cross-disciplinary perspectives and campus-wide best practices.

Throughout the project, the Guiding Team received technical assistance from Change Matrix’s Adaptive Change Specialists, who provided consultation on inclusive leadership, data interpretation, and systems change. This partnership supported the School in applying national models of equity-focused organizational change to the local context.

### Overall approach

The SPH used a multi-method, data-driven approach to identify barriers to equity, understand stakeholder perceptions and experiences, and design targeted interventions and processes. Initial data collection occurred using three primary strategies:

- A School-wide Climate Survey
- Listening Sessions with internal stakeholders
- Tenure and Promotion (T&P) Focus Groups with historically excluded faculty across campus.

Findings from all data sources were synthesized into a unified set of priority themes, which directly informed the design of all subsequent interventions, including faculty/staff workshops, the School-wide Community Agreement, and the Academic Fellowship (AEF) program. This data-to-action approach guided both implementation and ongoing refinement of activities and programs.

### Climate survey

In August–September 2022, the SPH administered an anonymous Climate Survey to faculty, staff, students, and recent alumni. The instrument was adapted from surveys previously used in the Colleges of Law, Education, and Communication & Fine Arts and included branching items tailored to each respondent group. Survey domains included perceptions of inclusion and belonging, equity in policies and practices, and experiences of support within the SPH.

A total of 88 respondents (19% of 457 invited) completed the survey. Respondents represented faculty, staff, graduate and undergraduate students, and alumni. Demographic information was collected to contextualize findings while maintaining confidentiality. Survey results provided a baseline for identifying priority areas and informed the design of subsequent qualitative data collection.

### Listening sessions

Between September and October 2022, nine facilitated listening sessions were conducted to gather deeper qualitative insights into experiences of inclusion, mentorship, academic support, and institutional climate.

- Four sessions were facilitated by colleagues from the College of Education and included:

1. Non-tenured faculty and postdoctoral fellows,
2. Tenured faculty,
3. Staff, and
4. The Dean’s Office and Guiding Team (*n* = 32 total).

- Four additional sessions were conducted by the Principal Investigator with students across all SPH programs (BSPH, MPH/MS, MHA, and PhD; *n* = 19).
- One session, conducted by the Principal Investigator, was held virtually with alumni (*n* = 8).

Participants were invited to share experiences related to belonging, mentorship, barriers to success, and perceptions of the School’s climate. Demographic information was collected to ensure representation of historically excluded groups and to contextualize themes. Listening session questions are provided in Supplementary Appendix I.

### Tenure and promotion focus groups

To examine structural barriers affecting faculty advancement, two focus groups were conducted with historically excluded faculty (*n* = 19) from colleges across the University. SPH faculty were intentionally excluded to avoid conflicts of interest and ensure candid discussion. The focus groups were conducted by a faculty colleague in the College of Education. Participants provided feedback on challenges related to mentorship, workload, recognition of community engaged scholarship, and transparency in T&P processes. These focus groups were a core component of the data gathering strategy and informed recommendations for policy review and faculty support structures. Focus group questions are provided in Appendix II.

### Data analysis

Quantitative survey data were summarized descriptively. Qualitative data from listening sessions and focus groups were analyzed using an inductive thematic approach. Team members independently reviewed notes and transcripts to generate initial codes, which were iteratively refined through discussion and consensus. A final set of themes was applied across data sources to identify shared patterns and differences across shareholder groups. Findings were then mapped to potential interventions, which guided the development of faculty/staff workshops, the SPH Community Agreement, and the Academic Equity Fellowship Program.

## Activities

### Faculty/staff workshops

We implemented three lunch-and-learn workshops focused on cultural humility and inclusive practices for UofM SPH faculty and staff (6). Workshops were designed to address priority themes identified through the data collection process, with a focus on actionable strategies to improve workplace climate, mentorship, and community-building among faculty, staff, and students.

Each workshop was designed to respond to these themes. For example, to cultivate a respectful environment, a session on emotional intelligence addressed the ability to recognize, understand, and manage our emotions and the emotions of others, which is useful in managing conflicts/interactions with colleagues. A Community Agreement session translated climate concerns about belonging and interpersonal respect into shared behavioral commitments. The resultant Community Agreement was designed to hold members accountable and to promote a safe, respectful environment. The training was provided by Guiding Team colleagues from the Law School, College of Education, and College of Communication & Fine Arts (Table 1).

**Table 1:** Mapping of data themes to workshop content.

Identified theme	Data source	Workshop response
Respectful workplace	Survey + listening sessions	Community agreement development
Provide training/mentorship	Survey + listening sessions	Development of mentorship standards
Cultivate community	Listening sessions	Development of informal social events

Attendance averaged 24 participants per session (range 16–27). Participants included faculty (59%), staff (30%), and administrators (11%). Demographic characteristics broadly reflected SPH composition; historically excluded and minoritized participants were represented in all sessions.

### Academic equity fellowship program

The Academic Equity Fellowship (AEF) program was launched in the 2022–23 academic year and was developed in response to student-identified needs for mentorship, professional connection, and skills to navigate academic culture. Climate survey and listening session data revealed that first-generation and historically excluded students often lacked access to informal knowledge networks that support advancement and success in graduate education (Table 2).

**Table 2:** Mapping of qualitative findings to AEF design.

Student-identified need	Program feature/component
Access to mentorship	Small cohort model
Financial constraints	Stipend support
Navigating academic norms	Professional development sessions
Sense of isolation	Community-building dinners
Advocacy skills	Self-advocacy workshops

## Program development

Qualitative themes were mapped to fellowship components as shown below.

The 6-session AEF Program introduced participants to frameworks, tools, and strategies that promote academic equity within public health and beyond. The workshops were designed to enable students to navigate the academic profession effectively while exploring how their personal narratives and professional goals intersect with community impact. The workshop series was designed to help Master’s students prepare for doctoral education and guide current doctoral students in their academic and leadership trajectories.

Sessions were developed and led by two doctoral candidates. Using feedback assessments to qualitatively evaluate the sessions, the iterative process was used in selecting future topics according to the Fellows’ expressed needs.

The program format and procedures were developed to accommodate student priorities and concerns: (1) sessions were conducted prior to evening classes, at a time convenient for students; (2) each AEF participant received a stipend of $600 ($100 for each of the six sessions); and (3) fellows shared a dinner together during each session.

### Participant demographics

Across two cohorts (*n* = 35 total fellows):

- 69% reported low-income backgrounds
- 63% identified as first-generation students
- 89% identified as from racially or ethnically minoritized groups
- 71% identified as female; 6% identified as non-binary
- 40% were master’s students and 60% doctoral students

While the program prioritized students considering academic pathways, participation was not restricted to those pursuing faculty careers. The rationale was that skills in leadership, advocacy, and navigating institutional structures are broadly applicable across public health careers.

### Outcomes

In the final session of the program, the Fellows were asked to reflect on the series, identifying key takeaways and actionable steps. The reflection could be in the form of a brief paper, or a poem, or other creative modality. In addition, two focus groups were conducted with the Fellows who participated in Year 1 (2022–23) to determine the perceived benefits of the program and to identify areas for program improvement. Reflections and focus groups highlighted:

- Increased confidence navigating academic systems
- Stronger peer networks
- Greater clarity about career pathways
- Enhanced sense of belonging
- Development of self-advocacy skills

Opportunities for improvement included longer and more frequent sessions; broader involvement (e.g., undergraduates, across departments, other universities, external organizations); increased financial support; and systems reform to provide feedback for improvements within the SPH. These findings suggest the fellowship program functioned as both a professional development and retention support strategy. Excepts from Reflection Papers are provided in Appendix III.

## Key findings

### Progress over the period of funding (2021 to 2025)

Over the funding period, the SPH implemented a series of data-informed strategies to strengthen inclusion, belonging, and academic support structures. Progress reflects both programmatic activities and institutional integration of equity-oriented practices.

#### Key accomplishments included

- Cross-campus partnerships: Established sustained collaborations with administrators and faculty from other colleges who brought prior experience in inclusive academic practices. These partnerships broadened institutional learning and supported cross-disciplinary knowledge exchange.
- Multi-method data collection: Implemented repeated climate surveys, listening sessions, and focus groups to identify priorities and monitor change over time.
- Faculty and staff development: Delivered ongoing inclusion-oriented workshops grounded in stakeholder feedback designed to strengthen mentorship, communication, and community norms.
- Community Agreement: Developed a school-wide agreement that articulated shared expectations for respectful engagement and collective accountability.
- Academic Equity Fellowship (AEF): Implemented two fellowship cohorts (Year 1: *n* = 18; Year 2: *n* = 17) to support first-generation and historically excluded graduate students through mentorship, professional development, and community-building.
- Student perceptions of climate: Annual student surveys showed increased positive perceptions of SPH commitment to a diverse educational community (86% agreement in 2023; 95% in 2025).
- Strategic plan integration: Embedded belonging and inclusion priorities into the SPH strategic plan with measurable indicators for monitoring progress.

These accomplishments reflect incremental but meaningful shifts toward a more supportive academic environment.

### Improvements noted in three years of climate survey results (2022–2025)

Although the perception-based trends exhibited in Table 3 below suggest positive movement, they should be interpreted cautiously. Improvements in reported climate may reflect increased awareness, engagement, or short-term effects of programming rather than sustained structural change. As such, these findings are best understood as indicators of progress rather than definitive evidence of long-term institutional transformation.

**Table 3:** SPH climate trends.

Survey item	2023 (*n* = 88)	2024 (*n* = 76)	2025 (*n* = 181)
“The School of Public Health has a welcoming climate or atmosphere”	95%	90%	95%
“I feel personally included in the School of Public Health”	84%	85%	86%
“The School of Public Health has made creating a diverse educational community a priority”	86%	90%	95%

### Lessons learned

Several key lessons emerged from this initiative:

- Take advantage of the resources offered. RWJF provided a wealth of resources and technical assistance. We made a conscious effort to utilize their expertise and support.
- Cross-disciplinary learning strengthened our initiative. We were fortunate to have colleagues at the UofM from whom we could learn. Collaborating with colleagues from other academic units accelerated learning and reduced the need to “reinvent” strategies
- Leadership support is essential. The support of administrative leadership was critical in legitimizing our efforts and ensuring sustainability. Their involvement signaled that this work was a priority.
- Community-building requires intentional design. Belonging does not emerge automatically; it must be cultivated through structured opportunities for connection, mentorship, and dialogue.
- Commit to sustainability. Lasting impact depends on embedding practices into structures, policies, and culture so the work continues beyond a single project or funding cycle.
- Progress is iterative. Cultural change occurs gradually. Regular reassessment allows institutions to adapt strategies rather than rely on one-time interventions.
- Celebrate successes. Recognizing and celebrating milestones – both large and small – helped maintain momentum, reinforced commitment, and built a sense of ownership.

### Implementation and sustainability

From the outset, sustainability was treated as a central goal rather than a final step. The SPH worked to integrate equity-oriented practices into routine structures and processes.

Academic equity and belonging have been incorporated into:

- New faculty orientation: Introduction to community norms, mentorship expectations, and teaching resources.
- New staff orientation: Emphasis on respectful workplace practices.
- New student orientation: Early exposure to belonging principles and available support networks.
- Committee on Faculty Affairs and Development: Offering ongoing professional development related to mentorship and inclusive practices.
- SPH strategic plan: Inclusion and belonging are explicitly reflected in goals, values, and evaluation metrics as well as in plans for recruiting, promoting, and retaining of the School’s faculty, staff, and students.
- Annual Climate Survey for Students, Staff, and Faculty: Instituted as a mechanism for accountability and continuous improvement.
- SPH updated syllabus format stating our School’s “responsibility to promote a safe, diverse and sensitive learning environment that respects the rights, dignity, and well-being of all students, faculty, and staff.”

By embedding these elements into existing structures, the SPH aims to maintain momentum beyond the grant period. Future sustainability efforts will focus on sustaining the AEF program, strengthening faculty mentorship capacity, reviewing policies that affect advancement, and continuing to monitor climate trends.

## Discussion

This initiative demonstrates how a data-guided, participatory approach can support institutional culture change in academic public health.

Several mechanisms may explain the observed changes. First, visible leadership support and integration of equity goals into the strategic plan likely signaled institutional commitment, increasing trust and engagement. Second, structured opportunities for interaction – such as workshops and AEF program – created spaces for relationship-building and skill development that are often missing in academic settings. Third, the explicit use of stakeholder data to inform action may have enhanced credibility and responsiveness, reinforcing participants’ sense that their experiences were being heard and addressed.

The most effective elements of this initiative appear to be those that combined skill-building with community-building. For example, workshops translated abstract principles of inclusion into concrete practices, while the AEF program addressed gaps in mentorship and informal knowledge sharing. These findings suggest that similar approaches may be transferable to other academic settings, particularly institutions that can leverage cross-campus expertise, engage leadership, and embed initiatives within existing structures.

We understand and believe that an inclusive environment fosters innovation, creativity, and academic excellence. At the same time, we recognize that equity initiatives in higher education are increasingly shaped by complex political dyanamics in which diversity-focused efforts may be scrutinized or contested. This context may influence both how initiatives are implemented and how they are framed, often requiring alignment with institutional priorities such as academic excellence, student success, and institutional effectiveness. While this alignment can broaden support, it may also constrain the scope of structural change efforts. Navigating this tension is an important consideration for institutions seeking to advance equity in the current environment.

### Limitations

While this initiative yielded meaningful insights and programmatic progress, several limitations should be acknowledged:

- Participation rates, while typical for climate surveys, limit generalizability
- Self-selection bias may have influenced listening sessions
- Outcome measures focused largely on perceptions rather than long-term retention or advancement

Future work should include longitudinal metrics such as student retention and performance, faculty promotion outcomes, and recruitment trends to better assess sustained impact.

## Conclusion

*Transforming Academia for Equity* at the UofM SPH illustrates our commitment to support ASPPH’s Strategic Plan 2030. We recognize that meaningful progress toward inclusion and belonging requires sustained, multi-level effort. While improvements in climate perceptions are encouraging, continued attention to faculty diversity, mentorship quality, and structural policy review remains essential.

## Data Availability

The datasets presented in this article are not readily available because data pertaining to the School of Public Health will not be released. Requests to access the datasets should be directed to mlevy@memphis.edu.
