# Supplementary material for: Transforming academia for equity: implementation at the University of Memphis
Source: Front Public Health. 2026 May 22;14:1690688. doi: 10.3389/fpubh.2026.1690688 (PMC13236637; doi:10.3389/fpubh.2026.1690688)

**APPENDICES**

**Appendix 1**

Listening Session Questions

1. Let’s think about inclusion – what comes to mind when you hear these terms?
2. How would you **currently describe** inclusion in School of Public Health?
3. What should we **target** to make a difference around inclusion as we enact our Strategic Plan in the SPH?
4. Imagine it is three years from now and we have been intentional and focused about enacting our Strategic Plan, what would you **see as evidence**in the SPH that verifies we are driven by inclusion?
5. What should we add to our vision?

**Appendix II**

Tenure and Promotion Focus Group Questions

1. How would you describe the academic climate related to equity in general at the University of Memphis?
2. Have you encountered any barriers or obstacles to academic equity at UofM?
3. Are there any policies or practices that have served as obstacles to your professional development or advancement? (e.g., recruitment? mentoring?)
4. What about tenure and promotion? Have you experienced any obstacles?
5. Are there any actions or policies in your college/school that have facilitated your academic success/tenure and promotion?
6. What recommendations do you have that would support academic equity at the University of Memphis? What actions should be taken?

**Appendix III**

**Three Excepts from AEF Reflection Papers (what they have learned and embodied):**

*1. “I am so happy to be able to be a part of this fellowship because it has given me the voice I need to stand up for myself. I have never felt so much pride in where I come from and can say proudly that I am from South Memphis. The lessons taught throughout these several weeks have made me realize that I am needed to improve my community. It has helped me recognize that I am a gem, and my ability to pursue a doctorate is remarkable. I have also been compelled to call out inequities and injustices and not be afraid because I must be present for those voices of encouragement still rooting for me.”*

*2. “We do not choose the circumstances or the way people treat us because of the groups we fall in. We do control how we allow the behavior to affect us and how we treat the people who has done harm to us. I am grateful for the opportunity to have a voice for myself. I will use the tools given to me and pass them along to others. I think that having a space to be seen and represented is important and supports the academic success of minority students.”*

3. REFLECTION PAPER: A POEM

*It was a time to get away and learn.*

*A time to breathe.*

*A time to stretch beyond ourselves.*

*A time for meditation.*

*A time to share our experiences.*

*A time to get full of new knowledge.*

*A time for communing.*

*A time for healing and growth.*

*This journey brought about new ways to navigate our time in this space. And that we’re not alone here. We have each other to walk with and support.*

*It was a time for our voices to be heard and to demand to be seen.*

*It was a time when we realized our humanity in this space as individuals and collectively.*

*It was a time to meet and discover all the tools we would need to be successful beyond this place.*

*It was a time to advocate for those that couldn’t advocate for themselves or felt invisible on their journey.*

*It was a time to understand that compassion and love are needed to make a difference, a change, or just simply recognize and understand someone else’s experiences.*

*Thank you, Academic Equity Fellows program for this experience to learn and grow. My life is richer because I’ve experienced others’ stories, and gained tools that will make my journey here better.*

*What a time we had!*

**Appendix IV**

**Climate Survey Results (2023-2025)**


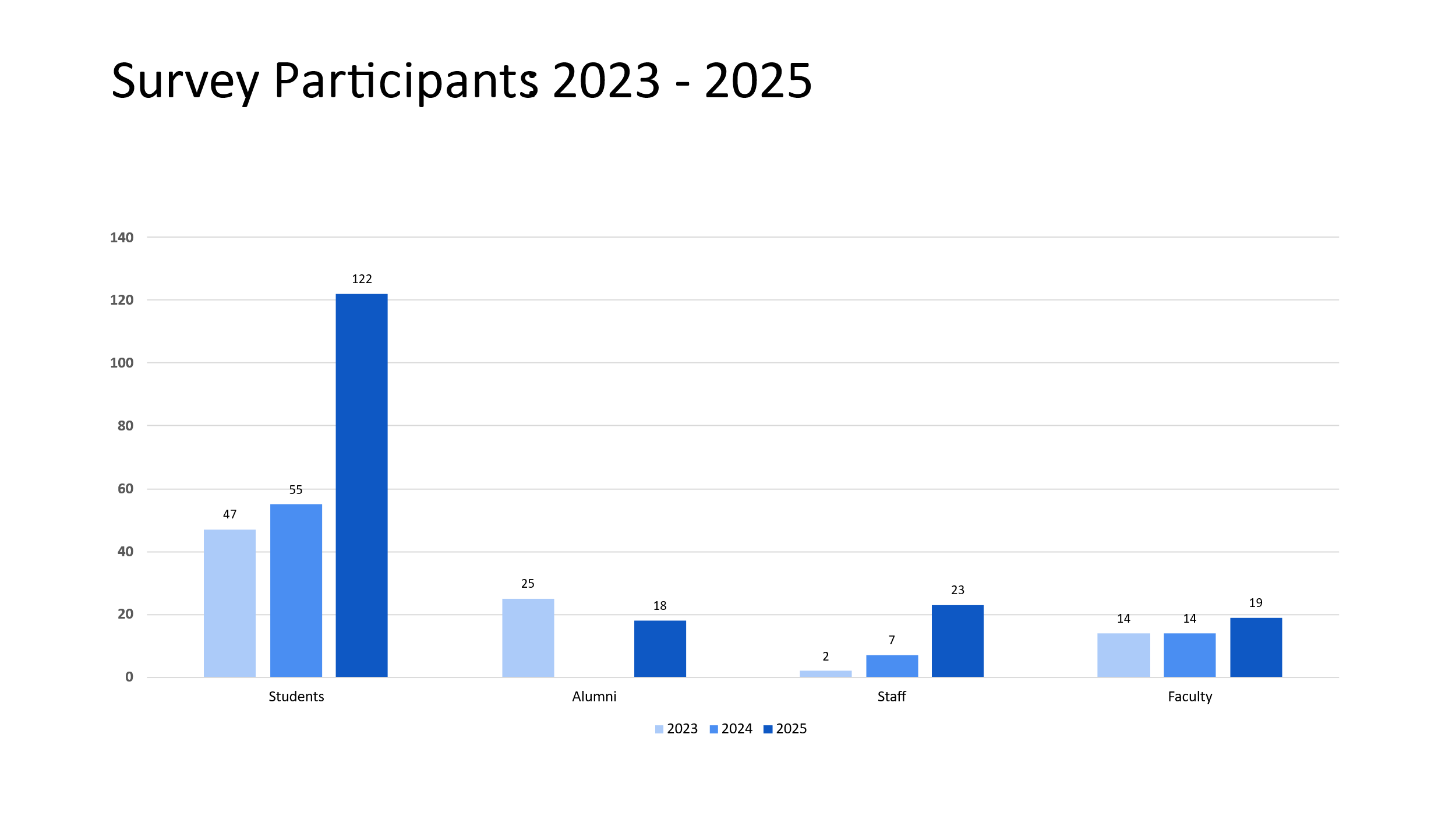


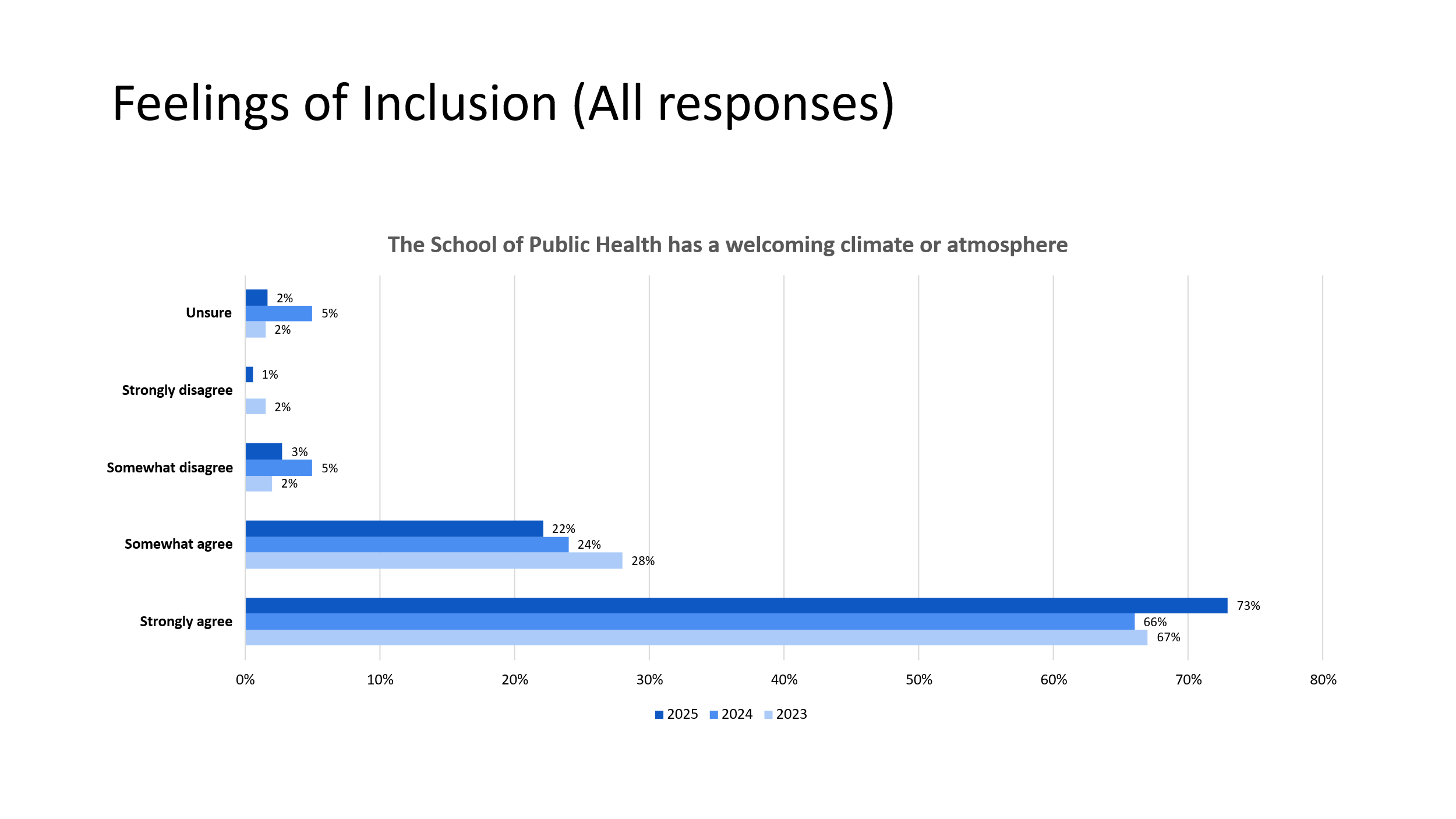


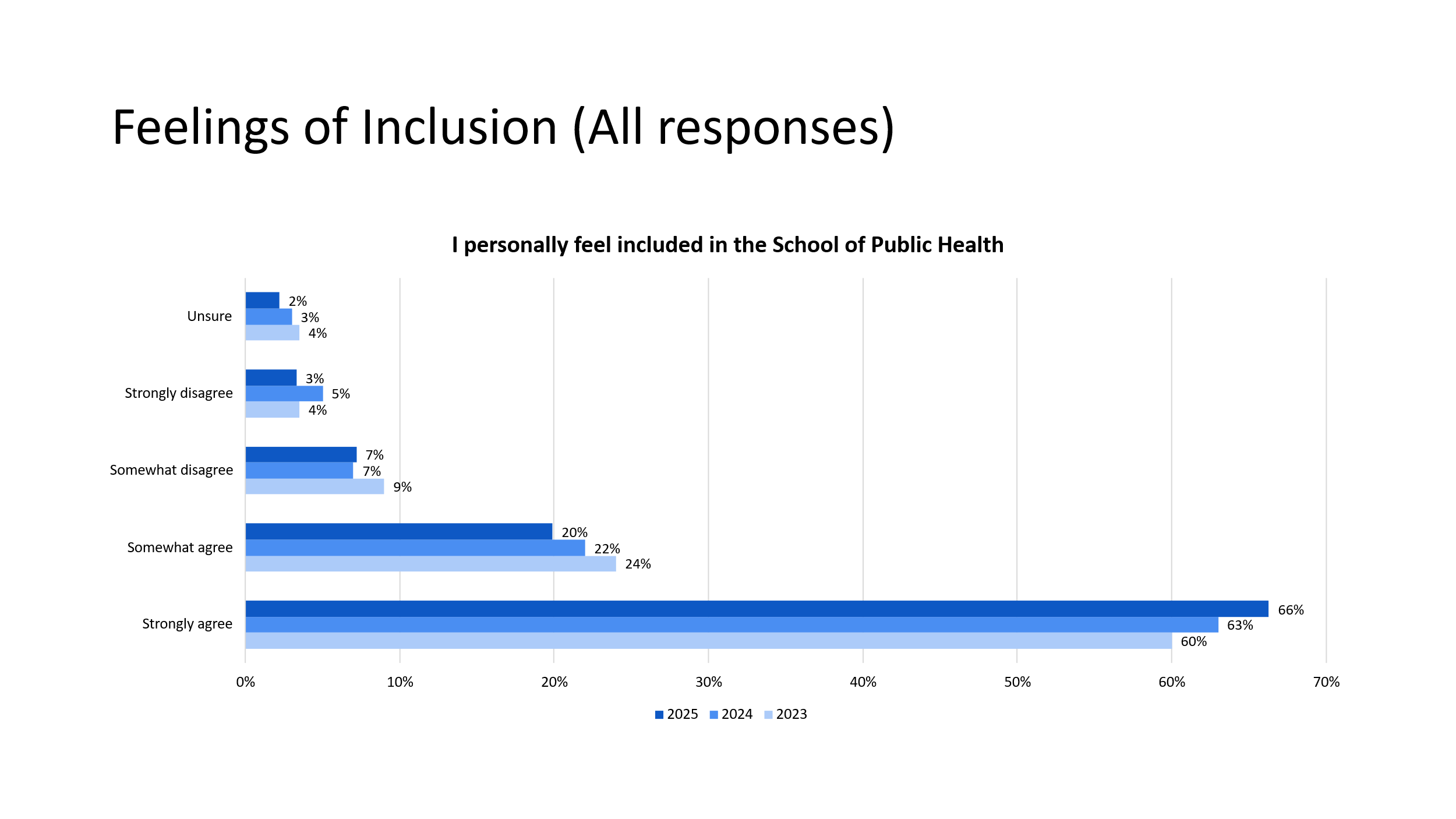

Supplement: Supplementary file 1 [file Data_Sheet_1.docx]
